# Global Studies of the Host-Parasite Relationships between Ectoparasitic Mites of the Family Syringophilidae and Birds of the Order Columbiformes

**DOI:** 10.3390/ani11123392

**Published:** 2021-11-27

**Authors:** Katarzyna Kaszewska-Gilas, Jakub Ziemowit Kosicki, Martin Hromada, Maciej Skoracki

**Affiliations:** 1 Department of Animal Morphology, Faculty of Biology, Adam Mickiewicz University, 61-614 Poznań, Poland; skoracki@amu.edu.pl; 2 Department of Avian Biology and Ecology, Faculty of Biology, Adam Mickiewicz University, 61-614 Poznań, Poland; kubako@amu.edu.pl; 3 Laboratory and Museum of Evolutionary Ecology, Department of Ecology, Faculty of Humanities and Natural Sciences, University of Presov, 080 01 Prešov, Slovakia; hromada.martin@gmail.com; 4 Faculty of Biological Sciences, University of Zielona Góra, 65-516 Zielona Góra, Poland

**Keywords:** Acari, biodiversity, quill mites, pigeons and doves, network

## Abstract

**Simple Summary:**

Mites of the family Syringophilidae (Acariformes: Cheyletoidea)—also called quill mites—are permanent and highly specialized ectoparasites of birds living inside the calamus of the various types of the feathers. In the present paper, we conducted a study focused on prevalence, host specificity, networks, and phylogeny of the syringophilid mites parasitizing on pigeon and doves (Columbiformes). We postulate that the Syringophilidae mites and Columbiformes bird system represent a model which can be used in a broader study of the relationship between hosts and parasites.

**Abstract:**

The quill mites belonging to the family Syringophilidae (Acari: Prostigmata: Cheyletoidea) are obligate ectoparasites of birds. They inhabit different types of the quills, where they spend their whole life cycle. In this paper, we conducted a global study of syringophilid mites associated with columbiform birds. We examined 772 pigeon and dove individuals belonging to 112 species (35% world fauna) from all zoogeographical regions (except Madagascan) where Columbiformes occur. We measured the prevalence (IP) and the confidence interval (CI) for all infested host species. IP ranges between 4.2 and 66.7 (CI 0.2–100). We applied a bipartite analysis to determine host–parasite interaction, network indices, and host specificity on species and whole network levels. The Syringophilidae–Columbiformes network was composed of 25 mite species and 65 host species. The bipartite network was characterized by a high network level specialization H2′ = 0.93, high nestedness N = 0.908, connectance C = 0.90, and high modularity Q = 0.83, with 20 modules. Moreover, we reconstructed the phylogeny of the quill mites associated with columbiform birds on the generic level. Analysis shows two distinct clades: *Meitingsunes* + *Psittaciphilus*, and *Peristerophila* + *Terratosyringophilus*.

## 1. Introduction

Knowing how many species inhabit Earth is among the most fundamental questions in science [1]. Despite often being neglected [2], one of the major components of biodiversity are parasites [3], comprising at least half of all species [4,5,6]; up to 75% of all interactions in food webs involve a parasitic species [3]. Many estimates of global species diversity of parasites are based on extrapolations of patterns of host specificity [2]; however, a contrast between the proportion that parasites comprise in local and global faunas suggests that parasites are most probably less host specific and more widespread than local scale studies suggest [6]. To get over such difficulties, it has been increasingly recognized that biotic interactions matter not only at local but also at regional, continental, or global spatial scales [7]. Thus, in determining the host specificity of parasites, more precise data on different parasitic taxa on different scales, from local to global, are crucially needed.

The networks can be useful to illustrate and analyze relationships and ecological interaction between any type of community [8]. Such analyses not only give a visual graph links between two trophic levels, but they also offer an opportunity to quantify indices such as parasite–host specificity and species richness in hosts, and they give a topological description of connectance, nestedness, or modularity [8,9].

The monophyletic Columbiformes are one of the oldest and the most diverse non-passerine clade of Neoaves, comprising more than 320 species grouped in one family Columbidae [10]. Pigeons and doves can be found in all zoogeographical regions except the high Arctic and Antarctic and adjacent islands [11]. Despite some studies suggesting as old as Cretaceous origin of Columbiformes [12], there is a major consensus that they diverged from other basal land bird clades in Eocene [13,14]; radiation of major extant lineages continued until the Miocene [15]. Thus, this group can serve as a good model for global studies of their ectoparasite richness, interactions, and specificity.

The quill mites belonging to the family Syringophilidae (Acariformes: Prostigmata) are highly specialized parasites of birds [16] that live inside the quills of feathers. These obligatory ectoparasites pierce the quill wall with extremely long stylet-like movable digits of chelicerae. Each stylet can be extended independently from the other, this movement occurs in the course of piercing the wall of quill and thus feed on live tissue fluids of their avian hosts [16,17,18,19].

Currently, the fauna of Syringophilidae comprises about 400 species belonging to 63 genera and associated with 27 bird orders [20]. However, Johnson and Kethley suggested that, considering their potential host richness, the number of quill mite species can reach as much as 5000 [21]. Most of the quill mites are either restricted to only one host species (monoxenous parasites) or adjusted to live in closely related hosts (oligoxenous parasites) [22,23]. However, the host specificity of syringophilids is still insufficiently investigated.

Until now, trophic interaction analyses and bipartite networks have been used for the description of quill mite associations with the following host groups: sunbirds (Passeriformes: Nectariniidae) [24] estrildids (Estrildidae) [25], and doves and pigeons (Columbiformes: Columbidae) [26]. However, more detailed studies of network indices such as connectance, modularity, nestedness and nest specificity of the quill mites from family Syringophilidae are still needed. The analysis of bipartite network metrics obtained for global quill mite–columbiform trophic interactions can shed more light on the architecture and relation between host and parasites.

Quill mite fauna associated with the members of Columbiformes comprises 25 quill mite species belonging to the following genera: *Meitingsunes* Glowska & Skoracki 2010, (8 species), *Peristerophila* Kethley, 1970 (6 species), *Psittaciphilus* Fain, Bochkov & Mironov 2000 (2 species), *Terratosyrinophilus* Bochkov & Pérez 2002, (2 species), and *Gunabopicobia* Skoracki & Hromada, 2013 (7 species), recorded from 65 bird species belonging to 22 pigeons and doves genera. The quill mites–columbiform fauna was studied by Hirst [27], Clark [28], Kethley [16], Lawrence [29], Casto [30,31], Bochkov and Mironov [32], Fain et al. [33], Bochkov and Perez [34], Bochkov and Fain [35], Bochkov et al. [36], Skoracki and Glowska [37], Nattres and Skoracki [38], Glowska and Skoracki [39], Skoracki [22], Skoracki and Dabert [40], Skoracki and Hromada [41], Kaszewska and Skoracki [42], and Kaszewska et al. [43,44,45,46].

### Historical Review of Quill Mite Genera Associated with Doves and Pigeons

Among five genera of the quill mites infested birds of the order Columbiformes, only one—*Gunabopicobia*—belongs to the subfamily Picobiinae. The first species of this subfamily—*Syringophilus zumpti*—was described by Lawrence in 1959 based on the material collected from *Streptopelia capicola* (type host) [29]. In 1970, Kethley moved this species to the genus *Picobia* in the subfamily Picobiinae. In 2011, Skoracki established a new genus *Neopicobia* and placed *P. zumpti* in its species content [22]. However, two years later, considering morphological details (hypostomal apex bumpy and apodemes I without thorn-like protuberances), it was moved by Skoracki and Hromada [41] a new monotypic genus *Gunabopicobia*. New host records as well as numerous new *Gunabopicobia* species were described by Kaszewska et al. [42] and Skoracki et al. [47].

Members of the subfamily Syringophilinae associated with columbiform birds belong to four genera. The revision of Syringophilidae conducted by Kethley in 1970 resulted in a description of a new genus *Peristerophila*. In 2002, Bochkov and Pérez [34] erected a new genus—*Castosyringophilus*—closely related to *Peristerophila*. They also moved *P. mucuya* Casto, 1980 to *Castosyringophilus*. Subsequently, new quill mites species (12 species) from both genera with new hosts records belonging to the orders Columbiformes, Accipitriformes, and Falconiformes were given in the following papers: Bochkov and Fain [35], Skoracki et al. [48]; Skoracki [22], Skoracki and Glowska [37]; Kaszewska et al. [43,46]. In 2020, Skoracki et.al. [49] carried out a comparative study of the ontogeny and morphological structures of the quill mites belonging to genus *Peristerophila*. The results of this study indicated that the females of this genus, which are characterized by the presence of the two morphotypes: homeomorph forms belong to the genus *Peristerophila* while the heteromorphy ones formerly referred to the genus *Castosyringophilus*. Therefore, considering the ontogeny, the genus *Castosyringophilus* was synonymized with the genus *Peristerophila*. In 2003, Bochkov and Fain [35] published the results of their taxonomic studies on syringophilid mites associated with parrots, and established the genus *Terratosyringophilus*. They moved the previously described species *Peristerophila longisoma* Casto, 1979 recorded from *Zenaida asiatica* to the genus *Terratosyringophilus*. To date, *Terratosyringophilus* is comprised of five quill mites species infesting both doves and parrots. The other genus known from doves and parrots is *Psittaciphilus*, described by Fain et al. in 2000 [33]. Originally, Fain and co-authors recorded it only from parrots, but later, Kaszewska and Skoracki [42] found two new quill mites species of this genus, *P. montanus* and *P. patagioenas*, on columbiform birds. The results of this study allowed to addition of another genus to Columbiformes–Psittaciformes hosts group. The last genus associated with columbiforms closely related to *Psittaciphilus* is *Meitingsunes* described by Glowska and Skoracki in 2010 [39]. Type species of this genus—*M. zenadourae*—was originally described as *Syringophilus zenadourae* by Clark 1964 [28]. In taxonomic revision of syringophilids, Kethley [16], moved this species to the *Peristerophila* genus. Finally, Glowska and Skoracki 2010 [39] based on morphological difference (apodemes I divergent, not fused to apodemes II) established a new genus—*Meitingsunes*—for this species. In 2011–2020, numerous taxonomic studies added five new quill mite species associated with birds from order Columbiformes. Currently, only one species of the letter genus, *M. caprimulgus*, has been recorded from another bird order, such as Caprimulgiformes [50].

Recent examples of studies on multispecies interactions at macroscales can be broadly grouped into two analytical approaches: analyses of species richness and ecological networks. The ecological network approach usually relies on observed interactions among multiple species at fine spatial resolutions [7].

Therefore, in this study, we focus on describing the richness, interactions, and measuring the specialization of syringophilid ectoparasites and their columbiform hosts in a global scale. Moreover, we reconstruct the syringophilid phylogeny at the generic level. Additionally, based on an earlier study, we summarize all taxonomic and locality records to create a worldwide distribution of the quill mites associated with birds from the order Columbiformes. We also discuss the host–parasite relationships between syringophilid species and columbiform birds.

## 2. Materials and Methods

In the present study, we re-examined the ornithological collections of the columbiform specimens housed in the Bavarian State Collection of Zoology, Munich, Germany (ZSM) and Museum of Natural History, Nairobi, Kenya (NMK). These bird collections have been previously used as donors of mite species described or recorded in the several published papers (see Skoracki and Dabert [40]; Skoracki and Glowska [37]; Glowska and Skoracki [39], Skoracki [22]; Skoracki and Hromada [41]; Skoracki et al. [47]; Kaszewska and Skoracki [42]; Kaszewska et al. [42,43,44,45,46]). We also analyzed the host specimens collected from frozen collections housed in several veterinary centers. Bird specimen was examined using a dissecting microscope and the infested quills were opened with a fine scalpel. From each bird specimen, we removed one wing covert, about 5 under-tail coverts, and about 10 contour feathers. Before mounting, mites were softened and cleared in Nesbitt’s solution at room temperature for three days [22], and then mites were mounted on slides in Hoyer’s medium.

### 2.1. Bipartite Networks and Statistics

**Figure 1:**
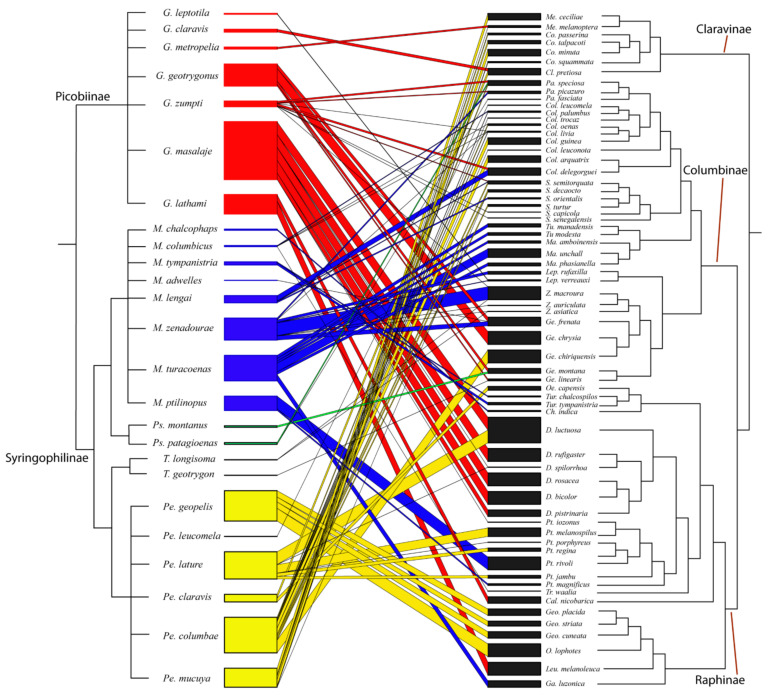
Bipartite network graph of interactions between quill mite species (**left**) and their doves and pigeons hosts (**right**).

The bipartite graph consists of rectangles representing compartment species and the width is proportion to the sum of interaction involving this species. Interacting species are linked by lines whose width is proportional to the number of interactions [51]. To visualize patterns in the studied host–parasite–ecological web, we used the ‘bipartite’ package available for R software [51]. To visualize bipartite networks we used functions plotweb (Figure 1) and visweb (Figure S1). For all host species recorded in earlier papers without information about prevalence, we gave score 1. Indices were calculated by using network-level and network-species functions available in bipartite packages.

We calculated the following bipartite index: network specialization (H2′) nestedness (N), connectance (C), and modularity (Q), to measured interaction on species-level we used species specialization metrics (d’). For this purpose, we prepared the matrices where quill mites species are in the rows (parasites) and the bird species (host) in the columns. ‘H2’ network-level measure of specialization, based on the deviation of a species’ realized number of interactions and that expected from each species’ total number of interactions [52]. Values of H2’ range from 0 to 1, where 0 indicates low specialization, while 1 suggests high specialization [53]. We also calculated the connectance, defined as the proportion of possible links observed in the network [54], ranged from 0 (low connectance in the network) to 1 to imply more connectance in the network. Nestedness measures how many interactions realized by specialists are a subset of those realized by generalists. The base metric of nestedness is the nestedness temperature T (0°–100°), which measures the departure from a perfectly nested interaction matrix [55]. For this study, we used a binary system, where metrics define as N = (100 − T)/100, with values ranging from 0 to 1 (maximum nestedness) [56,57,58].To calculate network modularity we calculate ‘likelihood’ implemented in computeModules in the bipartite library for R; this index is the same value as Q (or M), the modularity as given by Newman [59] or Guimerà & Amaral [60] and well known from QuanBiMo (Q) library [61], currently not supported. According to network permutation, we obtained 100 Q values (observed likelihood) [62] and compared them with 100 Q values coming from permutations for null models (null likelihood). To test a significant difference between the Q observed and Qnull values, we calculated the null.t.test (*p* < 0.05). For each quill mites species associated with doves and pigeons in the network, we calculated d’ index measured specialization at species level [52].

### 2.2. Prevalence

Descriptive statistics were computed using Quantitative Parasitology on the Web [63], with 95% confidence intervals (Sterne method).

### 2.3. Mite Phylogeny

In the cladistic analysis, we examined relationships at the generic level. All operational taxonomic units (OTUs) were represented by taxonomic species, i.e., type species for each genus. A free-living predator *Cheyletus eruditus* (Schrank) and quill-inhabiting predator *Metacheletoides numidae* Fain both belonging to the sister family Cheyletidae, were used as outgroups in the analyses. Because each particular syringophilid genus is represented by a single species in the present analysis, the character states appearing as autapomorphies represent true synapomorphies for genera.

A total of five OTUs representing all genera associated with columbiform birds, two taxa as the outgroup, and 26 non-additive and unordered morphological characters were included in our data matrix (data matrix and morphological characters are supplemented (Figures S2 and S3)). A detailed discussion of the morphological characters used in the present study is provided by Skoracki [22]; Skoracki et al. [47]. The matrix was done using NEXUS Data Editor 0.5.0 [64]. Analyses of character distribution on the tree were performed in WINCLADA [65]. Only unordered, qualitative, and unweighted characters were used in analyses. We applied a multistate contingent coding strategy [66], which is considered as the most useful among available approaches [67]. Following this strategy, characters with multiple states were interpreted as unordered and not modified into binary characters. Reconstruction of phylogenetic relationships was performed with PAUP 4.0 beta version for IBM [68] in conjunction with PRAP2 [69] to conduct a ratchet analysis (1000 iterations; 10 random cycles, collapsed zero-branches in effect; options are the default). Nodal support was evaluated by Bremer indices calculated with PRAP2. Analysis of character distributions, drawing, and editing of the trees was performed in TreeView 1.5.2. [70].

### 2.4. Visualization of Host Phylogeny

To visualize host phylogeny, a tree of the columbiform species was constructed based on a consensus avian phylogenetic tool available at http://birdtree.org/ (accessed on 5 March 2019) [71]. As the source of our consensus tree, we used the ‘Hackett All Species tree’ with 1000 randomly generated trees. The most credible tree was then determined using the tool TreeAnnotatorv1.8.2 in the software BEAST v1.8.2 [72]. The consensus tree was then graphically adjusted in FigTree v1.4.2 (Andrew Rambaut, University of Edinburgh, UK; http://tree.bio.ed.ac.uk/software/figtree/ (accessed on 5 March 2019)).

### 2.5. Host Specificity

Host specificity for particular mite species follows Caira et al. [73] and Skoracki et al. [47]. The division stands out monoxenous species (parasite infest single host species), oligoxenous (more than one host, but restricted to one genus), mesostenoxenous (more than one genus of hosts, but restricted to one subfamily), metastenoxenous (more than one subfamily of hosts but restricted to one order), and polyxenous species (more than one order). The common and scientific names of the birds follow Clements et al. [10]. Zoogeographic regions follow Holt et al. [74].

## 3. Results

A total of 772 individuals of pigeons and doves and belonging to 29 genera and 112 species were examined for the presence of quill mites belonging to the family Syringophilidae. Among them, 117 individuals representing 65 species had been infested by the quill mites belonging to the following genera *Meitingsunes* Glowska & Skoracki, 2010 (7 species), *Peristerophila* Kethley 1970 (6), *Psittaciphilus* Bochkov & Mironov, 2000 (2), (subfamily *Syringophilinae*), and *Gunabopicobia* Bochkov & Perez, 2002 (7) (subfamily Picobiinae) (Table 1 and Table S1).

In total, 22 out of 25 known quill mites species associated with Columbiformes birds were identified (*Terratosyringophilus geotrygonus*, *T. longisoma*, and *M. adwelles* were not found). Among non-infested columbid specimens, some taxa were examined for the presence of quill mites for the first time, for example: *Reinwardtoena reinwardtsi*, *Gymnophaps albertisii*, *Henicophaps albifrons*, and *Henicophaps foersteri*.

### 3.1. Prevalence Index Birds from Order Columbiformes

The index of prevalence (IP) of host species from Columbiformes order ranges from 4.2% to 100% (IP = 100 in 17 cases); however, the confidence intervals were wide and ranged from 0.2 to 100 (Table 2). In our material, 49 host species (239 individuals) were not infested by the syringophilid mites.

(1) IP 1–10% *Chalcophaps indica* (8.7%), *Columba livia* (8.7%), *Columba palumbus* (5%), *Columbina squammata* (6.7%), *Leptotila verreauxi* (4.2%), *Patagioenas picazuro* (6.2%), *Streptopelia orientalis* (9.1%), *Streptopelia semitorquata* (4.8%), *Turtur chalcospilos* (7%).
(2) IP 11–20% *Claravis pretiosa* (20%), *Columba delegorguei* (14.3%), *Columba oenas* (11.1%), *Columbina raucana* (13.3%), *Geotrygon linearis* (12.5%), *Geotrygon montana* (12.5%), *Leptotila rufaxilla* (20%), *Macropygia amboinensis* (16.7%), *Metriopelia melanoptera* (12.5%), *Patagioenas picazuro* (12.5%), *Patagioenas speciosa* (12.5%), *Patagioenas speciosa* (12.5%), *Ptilinopus magnificus* (11.8%), *Streptopelia semitorquata* (14.3%), *Streptopelia turtur* (13.3%), *Turacoena modesta* (20), *Turtur tympanistria* (16.7%).
(3) IP 21–30% *Columbina talpacoti* (25%), *Geotrygon montana* (25%), *Macropygia phasianella* (21.4%), *Oena capensis* (29.4%), *Turacoena manadensis* (25%).
(4) IP 31–40% *Geotrygon frenata* (33%), *Geopelia striata* (38.5%).
(5) IP 41–50% *Caloenas nicobarica* (50%), *Columba arquatrix* (50%), *Columba delegorguei* (42%), *Columba guinea* (50%), *Columbina minuta* (50%), *Gallicolumba luzonica* (50%), *Geopelia cuneata* (50%), *Geopelia placida* (50%), *Metriopelia ceciliae* (50%).
(6) IP 61–70 *Macropygia unchall* (66.7%), *Ptilinopus melanospilus* (66.7%).
(7) IP 100% *Ducula bicolor*, *Geotrygon chrysie*, *Geotrygon chiriquensis*, *Leucosarcia melanoleuca*, *Ocyphaps lophotes*, *Ptilonopus rauca*, *Zenaida macroura*.

In Table 2. We excluded the following examined but non-infested bird species: *Chalcophaps stephani* [N = 2], *Claravis mondetoura* [N = 2], *Columba rupestris* [N = 11], *Columbina cruziana* [N = 2], *Ducula aenea* [N = 4], *Ducula carola* [N = 2], *Ducula concinna* [N = 1], *Ducula finschii* [N = 2], *Ducula perspicillata* [N = 1], *Ducula zoeae* [N = 7], *Gallicolumba rufigula* [N = 1], *Geotrygon violacea* [N = 1], *Gymnophaps albertisii* [N = 2], *Henicophaps albifrons* [N = 1], *Henicophaps foersteri* [N = 1], *Leptotila cassini* [N = 1], *Leptotrygon veraguensis* [N = 1], *Macropygia magna* [N = 3], *Macropygia ruficeps* [N = 4], *Otidiphaps nobilis* [N = 1], *Patagioenas raucana* [N = 12], *Patagioenas cayennensis* [N = 18], *Patagioenas oenops* [N = 4], *Patagioenas subvinacea* [N = 3], *Ptilinopus bernsteinii* [N = 1], *Ptilinopus cinctus* [N = 6], *Ptilinopus coronulatus* [N = 4], *Ptilinopus ornatus* [N = 1], *Ptilinopus porphyreus* [N = 3], *Ptilinopus pulchellus* [N = 5], *Ptilinopus solomonensis* [N = 1], *Ptilinopus superbus* [N = 6], *Reinwardtoena reinwardtii* [N = 2], *Streptopelia picturata* [N = 1], *Streptopelia roseogrisea* [N = 3], *Streptopelia senegalensis* [N = 26], *Streptopelia tranquebarica* [N = 5], *Treron bicinctus* [N = 3], *Treron calva* [N = 7], *Treron capellei* [N = 3], *Treron curvirostra* [N = 8], *Treron delalandii* [N = 20], *Treron fulvicollis* [N = 4], *Treron olax* [N = 2], *Treron pompadora* [N = 7], *Treron sieboldii* [N = 5], *Treron sphenurus* [N = 3], *Treron vernanus* [N = 18], *Zenaida galapagoensis* [N = 8].

### 3.2. Host Specificity of the Quill Mites

Based on previously recorded host species, we classified all syringophilids associated with columbiform birds into the following host specificity groups (Table 3 and Table 4):

(1) Monoxenous parasites, including 8 species: *Gunabopicobia claravis*, *G. leptotila*, *G. metriopelia*, *Meitingsunes adewlles*, *M. chalcophas*, *Peristerophila leucomela*, *Psittaciphilus montanus*, *Terratosyringophilus geotrygonus*.
(2) Oligoxenous parasites, including 5 species: *Gunabopicobia geotrygoni*, *Meitingsunes ptilinopus*, *M. tympanistria*, *Psittaciphilus patagioenas*, *Terratosyringophilus longisoma*.
(3) Mesostenoxenous parasites, including 8 species: *Gunabopicobia lathami*, *G. masalaje*, *G. zumpti*, *Meitingsunes lengai*, *M. zenadourae*, *Peristerophila columbae*, *P. geopelis*, *P. lature*.
(4) Metastenoxenous parasites, including 3 species: *Meitingsunes turacoenas*, *Peristerophila claravis*, *Meitingsunes columbicus*.
(5) Polyxenous parasites, including only one species: *Peristerophila mucuya*.

### 3.3. Co-Infestation of the Quill Mites

The analysis of the host spectrum showed several various patterns of co-infestation with niche factor (quill mites occupying a different habitats) (Table 5):

(1) “*Syr-Pic*” (quill mite species belonging to the differential subfamily Syringophilinae or Picobiinae and inhabiting the same host species but different habitats.
  (i) Inhabiting niches: contour feathers (representatives of Picobiinae) and covert (representatives of Syringophilidae): *Guanabopicobia claravis* + *Peristerophila claravis* from *Claravis pretiosa*; *G. masalaje* + *P. lature* from *Ducula luctuosa* and *D. spilorrhoa*; *G. metriopelia* + *P. mucuya* from *Metriopelia melanoptera*, *G. zumpti* + *P. columbae* from *Streptopelia semitorquata* and *Patagioenas speciosa*.
  (ii) Inhabiting niche: contour feathers (Picobiinae) and under wing coverts (Syringophilidae): *G. geotrygoni* + *M. zenadourae* from *Geotrygon frenata*, *G. zumpti* + *M. zenadourae* from *Patagioenas picazuro*.
  (iii) Inhabiting niches: contour feathers (Picobiinae) and under tail coverts (Syringophilidae): *G. geotrygoni* + *Psittaciphilus montanus* from *Geotrygon, montana*, *G. leptotila* + *M. zenadourae* from *Leptotila verreauxi*.
  (iv) Inhabiting niches: contour feathers (Picobiinae) and rectrictres (Syringophilidae): *G. zumpti* + *M. lengai*.
(2) “*Syr-Syr*” (different quill mites species belonging to the same subfamily-Syringophilinae and occupying the same host species.
  (i) Inhabiting niches: secondaries and covert: Meitingsunes columbicus + Peristerophila columbae from Columba palumbus.

Moreover, in one sample, we observed quill mite species belonging to the same subfamily and inhabiting the same host species, and moreover occupying the same type of feathers. This pattern was found in *Peristerophila columbae* + *Psittaciphilus patagioenas* where both species occupied covert feathers of *Patagioenas speciosa* (Table 4).

### 3.4. Bipartite Network Analysis

The Columbiformes–Syringophilidae bipartite network (Figure 1) had high connectance (C = 0.90) and high specialization (H2’ = 0.93) with a high degree of nestedness (0.908). The comparison between H2’ and null model values, showed significant differences (mean for null model = 0.56; *p* = 0.0009271).

We also measured specialization on the species-level (d’). Quill mites specialization ranged between 0.20 and 1 (see Table 3 and Table 4).

(1) d’ 0.1–0.59: *M. adwelles* (0.2), *T. longisoma* (0.46), *T. geotrygonus* (0.5).
(2) d’ 0.6–0.99: *G. zumpti* (0.66), *P. montanus* (0.75), *P. patagioenas* (0.77), *M. columbicus* (0.78), *G. claravis* (0.78), *G. leptotila* (0.85), *P. lature* (0.86), *M. lengai* (0.9), *G. masalaje* (0.9), *M. zenadourae* (0.92), *G. geotrygoni* (0.92), *P. claravis* (0.92), *P. columbae* (0.95), *G. metriopelia* (0.98), *P. mucuya* (0.98).
(3) d’ = 1: *G. lathami*, *M. chalophaps*, *M. tympanistria*, *M. turacoenas*, *M. ptilinopus*, *P. geopelis*, *P. leucomela.*

The strength (thickness of connecting bar between parasites and hosts) of each interaction is representative of the number of interactions (prevalence). Each link corresponds to species interaction and represent quill mites genera: red—*Gunabopicobia*, blue—*Meitingsunes*, black—*Terratosyringophilus*, green—*Psittaciphilus*, yellow—*Peristerophila*. Host phylogeny based on Jetz et al. [68].

**Figure 2:**
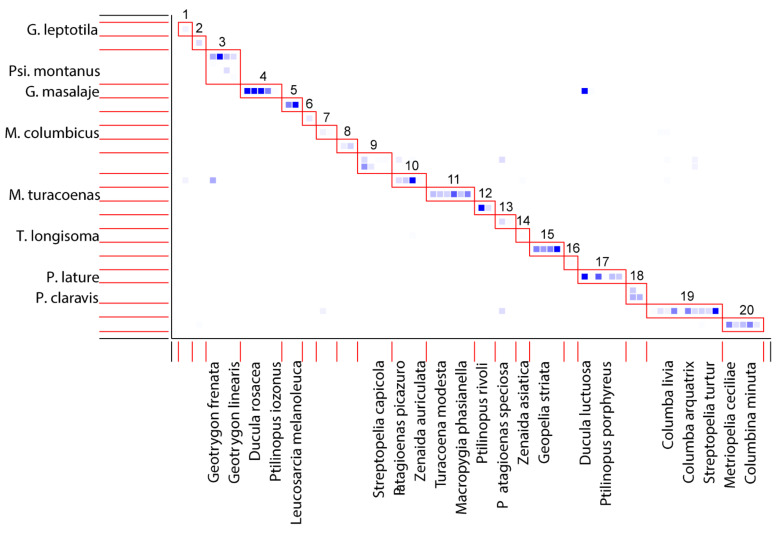
Modules of the quill mites–doves communities. Modules 1–20, generated for quill mites species and doves and pigeons. The intensity of the colors of the squares indicates the strength of the interaction, between particularly parasites species (vertical axis) and their hosts species (horizontal axis).

We registered a high modularity (likelihood = 0.83) with 20 modules. Modules were split to (A) single-host (quill mites associated with one host species), (B) multi-host (quill mites associated with more the one host species), and (C) multi-parasites (modules encompasses more than one quill mite species) modules (Figure 2).

- Single-host module: (1) *Gunabopicobia leptotila*—*Leptotila verrauxi*, (2) *Gunabopicobia metriopelia*—*Metriopelia melanoptera*, (6) *Peristerophila leucomela*—*Columba leucomela*, (14) *Terratosyringophilus longisoma*—*Zenaida asiatica*, (16) *Meitingsunes chalcophas*—*Chalcophaps indica*.
- Multi-host module: (4) *Gunabopicobia masalaje*—(*Ducula bicolor*, *Ducula rufigaster*, *Ducula rosacea*, *Ducula pistrinaria*, *Ptilinopus iozonus*); (5) *Gunabopicobia lathami*—(*Leucosarcia melanoleuca*, *Caloenas nicobarica*); (7) *Meitingunes columbicus*—(*Columba palumbus*, *Treron waalia*); (8) *Meitingsunes tympanistria*—(*Turtur chalcospilos*, *Turtur tympanistria*); (10) *Meitingsunes zenadourae*—(*Leptotila rufaxilla*, *Patagioenas picazuro*, *Zenaida auriculata*, *Zenaida macroura*); (11) *Meitingsunes turacoenas* (*Gallicolumba luzonica*, *Macropygia amboinensis*, *Macropygia phasianella*, *Macropygia unchall*, *Turacoena manadensis*, *Turacoena modesta*); (12) *Meitingsunes psittaciphilus* (*Ptilinopus magnificus*, *Ptilinopus rivoli*); (13) *Psittaciphilus patagioenas*—(*Patagioenas fasciata*, *Patagioenas speciosa*); (15) *Peristerophila geopelis* (*Geopelia cuneata*, *Geopelia placida*, *Geopelia striata*, *Ocyphaps lophotes*); (17) *Peristerophila lature* (*Ducula luctuosa*, *Ducula spilorrhoa*, *Ptilinopus jambu*, *Ptilinopus melanospilus*, *Ptilinopus porphyreus*, *Ptilinopus regina*); (19) *Peristerophila columbicus* (*Columba arguatrix*, *Columba guinea*, *Columba livia*, *Columba oenas*, *Columba trocaz*, *Columba leuconota*, *Geotrygon chiriquensis*, *Streptopelia decaocto*, *Streptopelia semitorquata*, *Streptopelia turtur*); (20) *Peristerophila mucuya* (*Columbina minuta*, *Columbina passerina*, *Columbina squammata*, *Columbina talpacoti*, *Metriopelia ceciliae*).
- Multi-parasite module: (18) *Peristerophila claravis*—*Gunabopicobia claravis Gunabopicobia geotrygoni*—*Meitingsunes zenadourae—Psittaciphilus montanus*—*Meitingsunes columbicus*; (9) *Gunabopicobia zumpti*—*Meitingsunes lenagi*.

### 3.5. Zoogeographical Distribution of Quill Mite Species Associated with Pigeons and Doves

**Figure 3:**
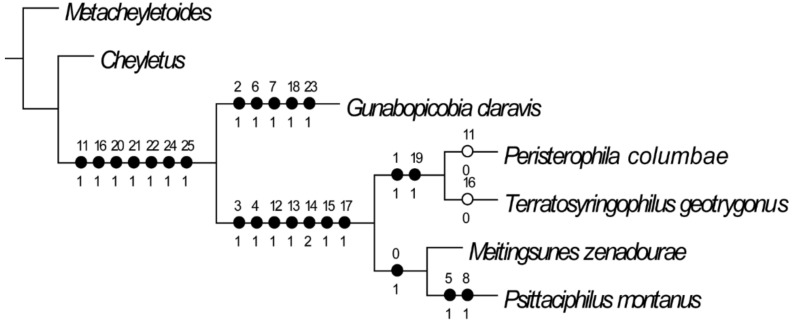
Phylogenetic tree of selected genera of Syringophilidae mites associated with Columbiformes birds.

Based on previous reports (see Table 1), we summarized the distribution of the Syringophilidae associated with birds from order Columbiformes. Quill mite species were recorded in hosts inhabiting the following zoogeographical regions: Neotropical, Nearctic, Panamanian, Palaearctic, Saharo-Arabian, Afrotropical, Oriental, Australasian, and Oceanian (Table 6, Figure 3). In particular regions, we noted the following genera with number of quill mites species:

- Neotropical: *Gunabopicobia* (5), *Meitingsunes* (2), *Peristerophila* (3), *Psittaciphilus* (2), *Terratosyringophilus* (1);
- Nearctic: *Meitingsunes* (1), *Peristerophila* (2), *Terratosyringophilus* (1), *Gunabopicobia* (1);
- Panamanian: *Psittaciphilus* (1), *Peristerophila* (2), *Gunabopicobia* (1);
- Palaearctic: *Meitingsunes* (8), *Peristerophila* (2), *Gunabopicobia* (1);
- Saharo-Arabian: *Peristerophila* (1);
- Afrotropical: *Meitingsunes* (4), *Peristerophila* (2), *Gunabopicobia* (1);
- Oriental: *Meitingsunes* (2), *Peristerophila* (4), *Gunabopicobia* (2);
- Oceanian: *Meitingsunes* (2), *Peristerophila* (1), *Gunabopicobia* (2);
- Australasian: *Meitingsunes* (2), *Peristerophila* (4).

Among all quill mites species, eight of them were only noted from one region: Neotropical—*Gunabopicobia metriopelia*, *G. claravis*, *G. leptotila*, *Meitingsunes adwelles*, *Psittaciphilus patagioenas Terratosyringophilus geotrygonus*; Afrotropical—*Meitingsunes tympanistria*; Australian—*Peristerophila leucomela*. Others quill mites species were recorded from more than one zoogeographical region:

- Neotropical + Nearctic + Palaearctic + Afrotropical: *Gunabopicobia zumpti*; *Meitingsunes zenadourae*; *Peristerophila claravis*;
- Neotropical + Nearctic: *Psittaciphilus patagioenas*;
- Palaearctic + Afrotropical: *Meitingsunes columbicus*, *Meitingsunes lengai*;
- Oriental + Oceanian: *Meitingsunes turacones*;
- Oriental + Australasian: *Meitingsunes chalcophas*;
- Oceanian + Oriental + Australasian: *Peristerophila lature*;
- Neotropical + Nearctic + Palaearctic + Afrotropical + Saharo-Arabian + Oriental: *Peristerophila columbae*.

### 3.6. Phylogenetic Analysis

**Figure 4:**
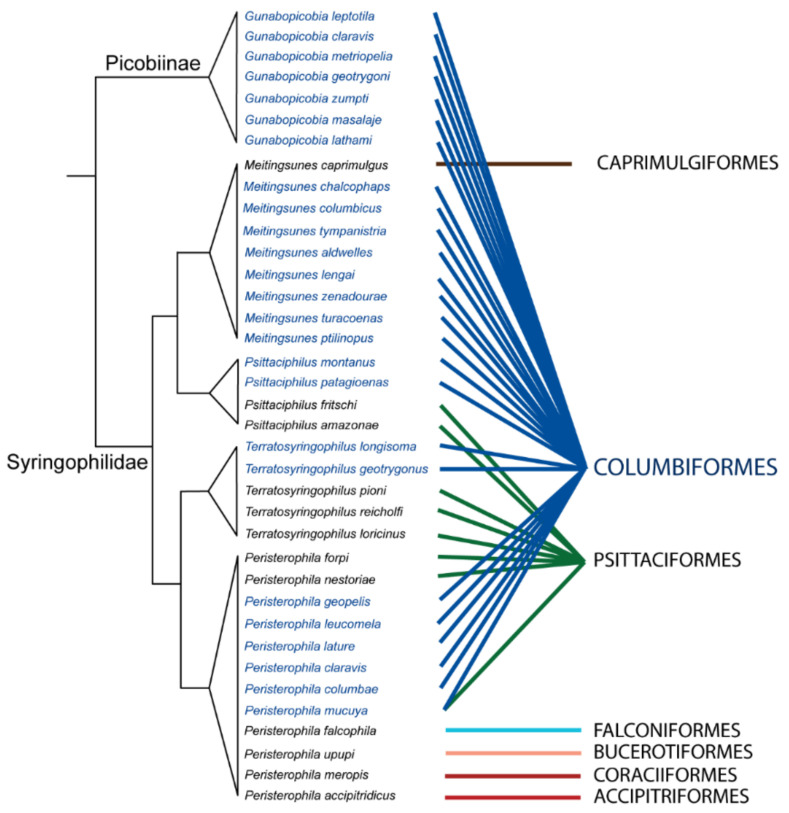
Phylogenetic tree of selected genera of Syringophilidae associated with differential hosts order.

The analysis under equal weights resulted in one most parsimonious tree (MPT) shown in Figure 4. Number of characters—26 (Figures S2 and S3), number of parsimony-informative characters—19, tree length (L) = 30, consistency index (CI) = 0.9, retention index (RI) = 0.9, rescaled consistency index (RC) = 0.8, homoplasy index (HI)—0.1, Goloboff-fits (G-fit) = −18.25.

The analysis shows that except the genus *Gunabopicobia* which represents subfamily Picobiinae, other syringophilinae genera form two distinct clades: *Peristerophila* + *Terratosyringophilus* (supported by synapomorphies: the presence of large finger-like protuberances on the hypostomal apex, presence of parallel apodemes I, and presence of dimorphic females) and *Meitingsunes* + *Psittaciphilus* (supported by synapomorphy: the presence of the constricted posterior end of the stylophore.

## 4. Discussion

The parasitological studies on quill mites of the family Syringophilidae and their hosts have a long history spanning over 140 years [16,22]. However, the extensive studies on this group of parasites started about 40 years ago, and investigation of a small fraction of the about 10,000 extant bird species recognized to date recording of more than 400 species of syringophilid mites arranged in 63 genera and two families [20].

The studies on the host–parasite relationship in the system composed of quill mites and the particular taxonomical groups of their hosts are still rare in the literature. Moreover, comprehensive research of the quill mite fauna on the host representatives of the whole bird order and considering mite species richness, host and habitat specificities, prevalence, and phylogenetic relationships have not been provided so far. Most of the previously published papers have focused on describing syringophilid fauna of the particular zoogeographical regions e.g., [22,23,77], or on taxonomical reviewing the different taxa (genus or subfamily) of quill mites e.g., [23,26,41,78]. Recently, however, there have been published a few studies examining the syringophilid fauna on the particular taxonomical host groups (e.g., passeriform genus *Estrilda* [25], sub-Saharan Nectariniidae [24], cuckoos [79]), with primary analyses of host–parasite relationships.

This paper focuses on analyses of the species richness and measuring specialization and interaction between syringophilid mites parasitizing columbiform birds in their natural host–parasite system.

### 4.1. Species Richness and Phylogenetic Relationship of Quill Mites Associated with Columbiform Birds

The fauna of quill mites associated with doves and pigeons encompasses 25 species belonging to the following five genera: *Meitingsunes*, *Peristerophila*, *Psittaciphilus*, *Terratosyringophilus* (subfamily Syringophilinae), and *Gunabopicobia* (subfamily Picobiinae) (see Table 1). Among them, only one—*Gunabopicobia*—is exclusively associated with columbiform birds and represented by monoxenous (3 species), oligoxenous (1), and mesostenoxenous (3) parasites. Thus, this genus is a perfect example of the host–parasite interaction where a supraspecific taxon of parasites is associated with one host order. This genus is known from hosts representing all columbiform subfamilies, i.e., Claravinae, Columbinae, and Raphinae. It was suggested by Kaszewska et al. [26] that mites of this genus could have started to parasitize the common ancestor in the Late Cretaceous (about 41 to 46 MYA) before their split on the particular subfamilies. Moreover, the Columbiformes are one of the oldest lineages of extant birds. A recent molecular study based on the complete mitochondrial genome suggests that the earliest radiation of the Columbidae occurred during the late Oligocene and continued diversification of the major clade in the Miocene [15]. However, older data suggest that the Columbiformes radiated from Eocene to Oligocene [13,14] or even from Early Eocene to middle Miocene [12].

Four genera of the syringophilines associated with columbiform birds have been previously assigned to the *Psittaciphilus*-generic-group [80]. In this study, we identified phylogenetically closely related clades *Meitingsunes* + *Psittaciphilus* and *Peristerophila* + *Terratosyringophilus*.

The genus *Meitingsunes* comprises nine described species where eight of them are exclusively associated with pigeons and doves infesting birds belonging to two subfamilies Columbinae and Raphinae (28 infested species in total) [45]. However, only one species of this genus, *M. caprimulgus,* has been noted from the phylogenetically distant clade of nightjars (Caprimulgiformes) [50]. Because birds belonging to the order Caprimulgiformes are extremely poorly examined (with only one host record), the status of *Meitingsunes* on nightjars is still unclear. The nightjars can represent real hosts for quill mites of this genus, or the single findings of *M. caprimulgus* can be an example of host-switching (e.g., from the columbiform host).

The genus *Psittaciphilus* includes four species found on representatives of Columbiformes (2 species) and Psittaciformes (2) [33,42]. On pigeons and doves, this genus infests birds of the genera *Geotrygon* and *Patagioenas*, which are also parasitized by members of the genus *Meitingsunes* mentioned above (e.g., *M. zenadourae* and *M. adwelles*).

The genus *Terratosyringophilus* includes three quill mites species found on parrots and two species noted from doves belonging to the subfamily Columbinae [31,34,35,37,81].

The *Terratosyringophilus* quill mites along with *Psittaciphilus* and partially *Peristerophila* (see below) have been found in birds from orders Columbiformes and Psittaciformes. The cases where both host orders are infested by mites belonging to the same genera can indicate the phylogenetically close relationship between these two bird orders. However, recent phylogenetic analysis does not confirm this hypothesis. It is commonly accepted that the lineage of doves and pigeons is a sister clade to sandgrouse (Pteroclidiformes) and mesites (Mesitornithiformes) [15,82,83,84]. At this moment, we cannot explain this multi-order infestation of the same genera of syringophilid mites. To resolve this problem, the molecular analyses of the quill mites phylogeny are needed as well as the studies on the host spectrum of the other symbionts parasitizing birds of these both orders.

The genus *Peristerophila* comprises 14 quill mites species and is the only genus that inhabits not only doves and parrots but also hosts belonging to hawks (Accipitriformes), falcons (Falconiformes), hoopoes (Bucerotiformes), rollers and bee-eaters (Coraciiformes) [43,46,48,75,85,86]. The *Peristerophila* mites associated with columbiform birds are recorded on hosts from all subfamilies, i.e., Columbinae, Raphinae, and Claravinae. Moreover, among all 30 species of syringophilines recorded on pigeons and doves, there is only one species—*Peristerophila mucuya*, representing polyxenous parasite—which is found on hosts belonging to the order Columbiformes and Psittaciformes (Figure 4).

### 4.2. Columbiform Hosts and Quill Mite Fauna

Our study of the quill mites associated with columbid birds was conducted on doves and pigeons representing all subfamilies, i.e., Columbinae, Claraviinae, and Raphinae. Based on material used for our research and records from previous publications, we estimated that the degree of species testing of Columbiformes ranged from 25% to 100% (for individual genera). In the subfamily Columbinae, regarding investigation degree of host species, more than 50% is in the following host genera: *Streptopelia* (52%), *Turacoena* (66%), *Zenaida* (85%), *Leptotrygon* (100%). In the subfamily Claravinae, we examined all currently recognized genera except monotypic genus *Uropelia*, *Claravis* (100%), *Columbina* (55%), *Metriopelia* (50%), *Paraclaravis* (50%). In the subfamily Raphinae, investigation degree more than 50% is in the following host genera: *Caloenas* (50%), *Chalcophas* (66%), *Geopelia* (60%), *Henicophaps* (100%), *Leptotrygon* (100%), *Leucosarcia* (100%), *Ocyphaps* (100%), *Oena* (100%), *Otidiphaps* (100%).

Considering high-level examination of columbiform birds under the presence of the quill mites, we suppose that the Syringophilidae fauna on the generic level has been fully explored. In the future, it would be worth intensifying research on the syringophilids inhabiting a single bird order. It will allow comparing our results with these ones conducted for other host orders. This approach allows for a better understanding of the parasite–host relationship as a whole. It would also be interesting to provide comprehensive studies on quill mite fauna associated with pacific island doves and pigeons. The future collection of the material from these regions will allow testing MacArthur and Wilson’s “the island theory” for quill mites. Additionally, future molecular studies on co-phylogeny also give important information about the relationships and evolutionary events between particular columbid and quill mite species.

### 4.3. Prevalence

The prevalence index provided details of the strength of the relationship between a particular host and parasites species. Our study has shown that the prevalence of infested birds by the quill mites ranges between 4.2% and 66.7%. However, for 17 hosts species, IP was equal to 100%, the confidence interval (CI) was wide, and this result can be the effect of the small sample size of studied host specimens.

The highest prevalences were detected in the previous studies for birds kept on the farms, e.g., domestic hen *Gallus gallus domesticus* infested by *Syringophilu bipectinatus* Heller, where IP was 75% (N = 1.500) [87] or for social species, e.g., house sparrow *Passer domesticus* infested by *Syringophiloidus minor* where IP was 82% (N = 492) [88]. For non-social birds, the prevalence index is much lower. It usually does not reach 50% (see works on prevalence among various passerine species (IP varies between 3.5% and 42.9%) [24,25,89,90,91,92,93]; phasianids (IP = 5.5–7.3%) [94,95,96]; parrots (IP = 7.7–20%) [97].

Both factors, the number of examined bird individuals and the number of examined feathers, play a crucial role in determining the real prevalence of infested hosts in the environment. In current and previous studies on prevalence, the used bird material was from various sources. The first source includes birds deposited in the museum collections (mostly dry bird skins and frozen or alcohol preserved specimens) e.g., [24,25]. The second source are birds examined during fieldworks (e.g, [18,48,88,90,92,93,95,98,99,100,101]) or kept in the zoological gardens [97] and farms [94,96,102].

It is obvious that syringophilid mites infest not all host specimens in nature and not all feathers, and to present the real IP, we should examine as many as possible bird individuals (taking into consideration their age, season, locality, etc.) and as many as possible feathers; see also [93,95,103]. However, samples collected from ornithological collections and from live birds are limited and allow sampling only a few feathers. Therefore, to minimize this limiting factor, we should continue studies on habitat specificity (see below).

### 4.4. Habitat Specificity and Multi-Infestation of Syringophilid Mites

The feather environment gives opportunities to inhabit various niches by ectoparasites and commensal species. However, the phenomenon of co-infestation remains poorly documented, especially for ectoparasites belonging to the family Syringophilidae. The first remark about multi-infestation was pointed out by Kethley [16]. He indicated that one host species or even one host individual may be infected by several syringophilid species inhabiting different types of feathers. Later on, Schmäschke et al. [104] presented the observation of co-infestation of two species, *Syringophilopsis turdi* and *Syringophiloidus* sp. on one the fieldfare *Turdus pilaris* (Passeriformes: Turdidae), and *Syringophilopsis kirgizorum* and *Syringophiloidus* sp. found on the greenfinch *Carduelis chloris* (Passeriformes: Fringillidae). Other examples of multi-infestations were described by Skoracki et al. [91]. In this paper, the authors recorded the following patterns of infestation with the notation of infested niches, e.g., *Torotrogla rubeculi* (habitat: secondaries) + *Picobia* sp. (habitat: contour feathers) on the European robin *Erithacus rubecula* (Muscicapidae); *Syringophilipsis kirgizorum* (primaries) + *Torotrogla gaudi* (secondaries) on the chaffinch *Fringilla coelebs* (Fringillidae); *Syringophiloidus presentlis* (secondaries) + *Picobia sturni* and *Aulonastus buczekae* (habitat: contour feathers) on the common starling *Sturnus vulgaris* (Sturnidae).

Until now, the multi-infestations by quill mites have been observed only in passeriform birds. However, our study described other cases of syringophilid multi-infestation on columbiform birds and showed that the phenomenon of co-infestation can occur more frequently. In total, we found 13 examples of co-infestation in different configurations. The most frequent cases of co-infestation were recorded for quill mites that inhabited the same host species but occupied differential niche—“factor niche”. For these cases, we observed two co-infestation patterns: (1) “*Syr-Pic pattern*”—quill mites belonging to two subfamilies Syringophilinae and Picobiinae occupying the same host individual or species; and (2) “*Syr-Syr pattern*”—quill mites belonging to the same subfamily, Syringophilinae. Currently, the pattern “*Pic-Pic*”, i.e., two species of picobiine mites on the same host species, was not observed. In members of the “*Syr-Pic pattern*”, representing two subfamilies, differences in morphology, life strategies, and niche preferences are clearly visible. For example, Picobiinae inhabit exclusively contour feathers while the members of Syringophilinae occur mainly inside the quills of secondaries, wing or tail coverts, rectrices; however, they are also occasionally found in contour feathers (Table 2 and Table 5). For the ”*Syr-Syr pattern*”*,* we observed a similar strategy of avoiding competition by occupying different feathers. However, for this group, we found two species, *Peristerophila columbae* and *Psittaciphilus patagioenas*, that infested the same host species and occupied the same niche—quills of wing coverts. Probably, this event could be an example of the horizontal transfer.

Niche separation among quill mites is a result of avoiding competition for the same microhabitat. According to the niche conception, the differential species cannot occupy the same niche (and use the same resources) because the advantage for one competitor will eventually drive others to extinction [105,106,107]. Finally, niche separation is the process of natural selection which drives competing species into using different hosts or different microhabitats [108].

Examples of niche separation are common and well documented for other ectoparasitic mites, e.g., mites from genus *Schizocarpus* infested *Castor fiber* [109] or feathers mites such as *Microspalax brevipes* and *Zachvatkinia ovata* associated with *Calonectris borealis* [110]. However, knowledge about competition and niche overlap phenomena for ectoparasites of the Syringophilidae is still unsuccessfully documented. The following examples of co-infestation in syringophilid groups provided in our study confirm the previous reports on the high degree of specificity of the quill mites to occupying niche. The observed preferences of syringophilids to colonize various types of feathers can result from the preferences to the specific parameters of the quills, such as the thickness of the quill wall and its volume. This hypothesis was proposed by Kethley [17], Casto [18], and Glowska et al. [111]. Moreover, recent studies by Grossi and Proctor [93] confirmed a strong correlation between quill volume and the average number of quill mites.

### 4.5. Bipartite Network of the Quill Mites–Doves Communities

The ecological network approach provides a lot of information about biological systems. Networks can be useful to illustrate and analyze the relationships and ecological interactions inside various types of communities [8]. Recently, an extensive study of an ecological network aimed to describe the character of mutualistic plant–animal interactions (pollination, seed dispersal, etc.) [5,56,112,113]. However, the network-thinking approach may also be useful in the study of the parasite ecology. Those analyses give a visual graph that illustrates links between two trophic levels, but above all, quantify indices such as host specificity in parasites and provide the topological description [5,9].

Network analyses were conducted for host–parasite systems, e.g., herbivorous insects–parasitoid food web [114] or tropical bats and their ectoparasitic bloodsucking flies [113]. Until now, bipartite analyses have been used for quill mites associated with the following host groups: sunbirds (Passeriformes: Nectariniidae) [24], estrildids (Estrildidae) [25], and doves (Columbiformes: Columbidae) [26].

In the present study, to describe the bipartite network, we used the following indices: connectance (C), nestedness (N), modularity (Q), and H2’. The values of these metrics provided information about: the number of interactions, the level of sharing partners, the degree of compartmentalization of the networks, and network-level specialization [52,115,116]. Our results confirm the hypothesis about the high specialization of syringophilid mites associated with pigeons and doves. We found strong specialization on both the network- and the species-level. The architecture of the quill mites-doves network was characterized by a high: connectance (C = 90), nestedness (N = 0.908), H2’ (H2’ = 0.93), and also with simultaneously high value of modularity (Q = 0.83) with 20 modules.

Recent studies of ecological networks have shown that the metrics such as nestedness, modularity, and connectance are correlated and depend on one another [57,58,117], which can be useful to understand the interaction between particular species in the network. One of the most important indices used to describe the quill mites-doves network was nestedness. We noted a high value of (N) = 0.908, close to 1. According to Bascompte et al. [56], the results close to 1 indicate a non-random community structure with a high level of diversity and complexity. Moreover, quill mites–doves communities were shown to have a highly modular structure. Modularity measures the tendency of a network to divide into modules (also called groups, clusters, or communities) [57]. It promotes stability by containing perturbations within a module, thereby constraining their spreading to the rest of the community [118]. In our networks, we found 20 modules, each of them had a strong interaction between species inside the modules. Some recognized modules (Figure 2) have more than one quill mite species, e.g., module number “3” had the highest number of quill mites species: *G. geotrygoni*, *M. zenadourae*, *P. montanus*, and *M. columbicus*. These multi-parasite communities interact with numerous hosts and probably can result from the phylogenetic relationship between particular quill mites and their hosts. The genera *Psittaciphilus* and *Meitingsunes* are sister clades (Figure 3) within subfamily Syringophilinae and share the same close relation to host species, while the genus *Gunabopicobia* is a separately evolutionary line. Moreover, those results suggest the structure of communities where competition for hosts can be expected. We observed another situation for modules where only one quill mite species has infested one host species. Those communities are represented, for example, by *Gunabopicobia metriopelia* associated with one host species, *Metriopelia melanoptera*. In this case, strong interaction with hosts species was observed.

The next indicator of complexity—connectance—was used in this study. The strong link between parasitic species and individual hosts (C = 0.90) observed in our research may be the result of non-random infestation.

The similar architecture of the bipartite network was presented in a study of ectoparasitic flies of the family Streblidae (Hippoboscoidea) and bat hosts from the tropical dry forest [113]. The authors of this study obtained structures similar to ours, such as high specialization (H2’ = 0.67), high modularity (Q = 0.7), but, contrary to the quill mites–doves nest, the authors found a low value of connectance (C = 0.30). The differential between C index can be related to sampled and network size. This relation was observed in the following networks: food webs (marine, estuarine, terrestrial), plant–pollinator, plant–herbivores–parasitoids in the forest [119,120,121]. However, some authors suggest that the connectance decreases when specialists are lost or generalists are gained [122,123]. In the quill mites–doves network, the proportion of specialized species is higher compared with the bat–fly network. Additionally, some analyses focusing on the conservation and protection of biodiversity suggest that the high C-value characterizes more stable communities, while low C-value can be an indicator of an ecological threat [122]. We hypothesize that the high C-value is observed in the stable and old hosts-parasites systems.

The Columbiformes and syringophilid mites have a long, common history. Quill mites have probably been associated with birds hosts for a very long time. Some studies based on the phylogeny of Syringophilidae and birds indicate that the quill mites of the family Syringophilidae could be associated with Neornithes birds around 66 million years ago or earlier [124]. Currently, the family Syringophilidae comprises about 400 species associated with birds from 27 orders. Most infested bird species belong to the clade Neoaves. However, quill mites species have also been found in Paleognathae (2 quill mite species), as well as Galloanserae (23 quill mite species) [20]. Considering the richness of parasites that inhabit modern birds, [124] suggested that their origins are not later than the Late Jurassic. Phylogeny analysis conducted by Skoracki et al. [78] showed that the mites on the earliest derivate branches are associated with birds of the advanced clade Neoaves. In contrast, genera associated with the earliest clades of extant birds, such as Tinamiformes (Palaeognathae) and Galloanserae (Anseriformes and Galliformes), are mosaically distributed in the core of the tree. On the other hand, ancestors of the quill mites could be associated with bird-like creatures before the K-Pg extinction event. Phylogeny analysis of parasitic mites from the superfamily Cheyletoidea (Acariformes: Prostigmata) showed that the Syringophilidae probably originated from a common ancestor with Cheyletidae, a predatory ancestor and inhabiting the litter of bird nests [124,125].

However, comparing the presented results with another host–parasites network is still unsatisfactory. The network-thinking approach used for the study of ectoparasites–hosts systems is limited. The most available research on bipartite networks was conducted on the mutualistic plant–pollinator food web. Moreover, we suggest that co-evolutionary analysis will be important to understand better the nature of the relationship between quill mites and doves.

## 5. Conclusions

The relation and interactions between host and parasites are still not well understood. We believe that this study focused on host specificity, prevalence, networks and evolutionary aspect has a particular role to identify the relation between host and parasites. The results of the presented study show that the quill mites belonging to family Syringophlidae and associated with pigeons and doves (Columbiformes) form stable and non-random communities.

The quill mites–doves bipartite has been characterized by a high value of nestedness, connectance, modularity, and H2’. We suggest that the observed network architecture in this study as well as high specificity and worldwide distribution of syringophlid mites is characteristic for: high host specificity systems with a long and common history.

## Figures and Tables

**Table 1:** Quill mite species of the family Syringophilidae parasitizing birds of the order Columbiformes with their distribution.

Quill Mite Species	Host Species	Host Subfamily	Distribution	References
Subfamily Syringophilinae Lavoipierre, 1953				
Genus *Meitingsunes* Glowska & Skoracki, 2010				
*M. aldwelles* Glowska & Skoracki, 2010	*Geotrygon frenata* * (Tschudi)	Columbinae	Neot. (Colombia)	[39]
*M. columbicus* Skoracki, 2011	*Columba oenas* * Linnaeus	Columbinae	Pala. (Kazakhstan)	[22]
“	*Columba livia* Gmelin	Columbinae	Pala. (Poland, Slovakia)	[22,45]
“	*Columba palumbus* Linnaeus	Columbinae	Pala. (Germany, Russia)	[22,45]
“	*Treron waalia* (Meyer)	Raphinae	Afro. (Cameroon)	[43]
*M. chalcophas* Kaszewska, Skoracki & Kavetska, 2016	*Chalcophas indica* * (Linnaeus)	Raphinae	Orie. (Indonesia: Timor) Aust. (Australia)	[44,45]
*M. ptilinopus* Kaszewska, Skoracki & Hromada, 2020	*Ptilinopus magnificus* * Temminck	Raphinae	Aust. (Australia)	[45]
“	*Ptilinopus rivoli* (Prevost)	Raphinae	Ocea. (Papua New Guinea)	[45]
*M. lengai* Kaszewska, Skoracki & Hromada, 2020	*Columba delegorguei* * Delegorgue	Columbinae	Afro. (Tanzania)	[45]
“	*Streptopelia orientalis* (Latham)	Columbinae	Afro. (Tanzania)	[45]
“	*Streptopelia semitorquata* Ruppell	Columbinae	Pala. (Kazakhstan, Kyrgyzstan)	[45]
*M. turacoenas* Kaszewska, Skoracki & Kavetska, 2016	*Gallicolumba luzonica* Scopoli	Raphinae	Orie. (Philippines)	[45]
“	*Macropygia amboinensis* (Linnaeus)	Columbinae	Ocea. (Papua New Guinea)	[45]
“	*Macropygia phasianella* (Temminck)	Columbinae	Orie. (Philippines, Indonesia: Java)	[45]
“	*Macropygia unchall* (Wagler)	Columbinae	Ocea. (Papua New Guinea)	[45]
“	*Turacoena manadensis* * (Quoy &Gaimard)	Columbinae	Orie. (Indonesia: Sulavesi, Nepal)	[44]
“	*Turacoena modesta* (Temminck)	Columbinae	Orie. (Indonesia)	[44]
*M. tympanistria* (Skoracki & Dabert, 2012)	*Turtur chalcospilos* (Wagler)	Raphinae	Afro. (Tanzania)	[40]
“	*Turtur tympanistria* * (Temminck)	Raphinae	Afro. (Togo, Tanzania)	[40,45]
*M. zenadourae* (Clark, 1964)	*Columba livia* Gmelin	Columbinae	Near. (USA: Texas); Afro. (N. Africa, Djibouti)	[30,39,45]
“	*Geotrygon frenata* (Tschudi)	Columbinae	Neot. (Colombia)	[45]
“	*Leptotila rufaxilla* (Richard, Bernard)	Columbinae	Neot. (Surinam, Argentina)	[45]
“	*Leptotila verreauxi* (Bonaparte)	Columbinae	Neot. (Colombia)	[45]
“	*Patagioenas picazuro* Temminck	Columbinae	Neot. (Paraguay)	[45]
“	*Zenaida asiatica* (Linnaeus)	Columbinae	Near. (USA: Texas)	[75]
“	*Zenaida auriculata* (Murs)	Columbinae	Neot. (Argentina)	[76]
“	*Zenaida macroura* * (Linnaeus)	Columbinae	Near. (USA: Maryland, Arizona, San Francisco)	[28,39,45]
Genus *Peristerophila* Kethley, 1970				
*P. columbae* (Hirst, 1920)	*Columba arguatrix* (Temminck)	Columbinae	Afro. (Kenya)	[46]
“	*Columba guinea* Linnaeus	Columbinae	Afro. (S Africa, Tanzania)	[46]
“	*Columba leuconota* (Vigors)	Columbinae	Orie. (Nepal)	[46]
“	*Columba livia* * Gmelin	Columbinae	Pala. (England, Macedonia, Poland, Turkey); Near. (Canada, USA); Orie. (India); Sa-Arab. (Iran)	[22,27,32,38,46]
“	*Columba oenas* Linnaeus	Columbinae	Pala. (Germany)	[46]
“	*Columba palumbus* Linnaeus	Columbinae	Pala. (Germany, England)	[46]
“	*Columba trocaz* Heineken	Columbinae	Pala. (Portugal)	[46]
“	*Geotrygon chiriquensis* Sclater	Columbinae	Pana. (Panama)	[46]
“	*Patagioenas speciosa* (Gmelin)	Columbinae	Neot. (Surinam)	[46]
“	*Streptopelia capicola* (Sundevall)	Columbinae	Afro. (Angola)	[46]
“	*Streptopelia decaocto* (Frivaldszky)	Columbinae	Sa-Arab. (Jordan)	[22]
“	*Streptopelia decipiens* (Hartlaub & Finsch.)	Columbinae	Pala. (Macedonia), Afro. (Tanzania)	[46]
“	*Streptopelia orientalis* (Latham)	Columbinae	Orie. (Japan)	[46]
“	*Streptopelia semitorquata* (Ruppell)	Columbinae	Afro. (Angola, Tanzania, D. R. Congo)	[46]
“	*Streptopelia tranquebarica* (Hermann)	Columbinae	Orie. (China)	[46]
“	*Streptopelia turtur* (Linnaeus)	Columbinae	Pala. (Germany, Greece, Hungary, Macedonia)	[46]
*P. claravis* (Skoracki & Glowska, 2008)	*Claravis pretiosa* * Ferrari-Pérez	Claravinae	Neot. (Bolivia, Colombia, Paraguay), Pana. (Panama)	[37,46]
“	*Oena capensis* (Linnaeus)	Raphinae	Afro. (Ethiopia, Sudan, Tanzania)	[46]
*P. geopelis* Kaszewska, Skoracki, Kosicki & Hromada, 2020	*Geopelia cuneata* (Latham)	Raphinae	Austr. (Australia)	[46]
“	*Geopelia placida* Gould	Raphinae	Austr. (Australia)	[46]
“	*Geopelia striata* * (Linnaeus)	Raphinae	Orie. (Indonesia: Celebes, Java, Sumatra)	[46]
“	*Ocyphaps lophotes* (Temminck)	Raphinae	Austr. (Australia)	[46]
*P. lature* Kaszewska, Kavetska & Skoracki, 2014	*Ducula luctuosa* * (Temminck)	Raphinae	Austr. (Australia)	[43]
“	*Ducula spilorrhoa* (Gray)	Raphinae	Austr. (Papua New Guinea)	[43]
“	*Ptilinopus jambu* (Gmelin)	Raphinae	Orie. (Indonesia: Sumatra)	[43]
“	*Ptilinopus melanospilus* (Salvadori)	Raphinae	Orie. (Indonesia: Mount Gade)	[43]
“	*Ptilinopus porphyreus* (Temminck)	Raphinae	Orie. (Indonesia: Java)	[43]
“	*Ptilinopus regina* (Swainson)	Raphinae	Orie. (Indonesia: Marina Isl.)	[43]
*P. leucomela* Kaszewska, Skoracki, Kosicki & Hromada, 2020	*Columba leucomela* * Temminck	Columbinae	Austr. (Australia)	[46]
*P. mucuya* Casto, 1980	*Columbina minuta* Linnaeus	Claravinae	Neot. (Paraguay)	[46]
“	*Columbina passerina* * (Linnaeus)	Claravinae	Neot. (Colombia, Surinam); Near. (USA)	[30,46]
“	*Columbina squammata* (Lesson)	Claravinae	Neot. (Brazil, Paraguay)	[35,46]
“	*Columbina talpacoti* (Temminck)	Claravinae	Neot. (Brazil, Surinam, Trinidad and Tobago); Pala. (Monaco)	[37,46]
“	*Geophaps plumifera* Gould	Columbinae	Aust. (Australia)	[35]
“	*Metriopelia ceciliae* (Lesson)	Claravinae	Neot. (Peru)	[46]
“	*Metriopelia melanoptera* (Molina)	Claravinae	Neot. (Argentina)	[37]
“	*Brotogeris versicolurus* ** Muller	Psittacidae	Neot. (Brazil)	[35]
“	*Psilopsiagon aymara* ** d’Orbigny	Psittacidae	Neot. (S. America)	[35]
“	*Trichoglossus haematodus* ** (Linnaeus)	Psittaculidae	Ori. (Indonesia)	[35]
Genus *Psittaciphilus*, Bochkov & Mironov, 2000				
*P. montanus* Kaszewska & Skoracki, 2018	*Geotrygon montana* * (Linnaeus)	Columbinae	Neot. (Brazil, Trinidad and Tobago); Pana. (Panama)	[42]
*P. patagioenas* Kaszewska & Skoracki, 2018	*Patagioenas fasciata* * (Say)	Columbinae	Neot. (Colombia)	[42]
“	*Patagioenas speciosa* (Gmelin)	Columbinae	Neot. (Surinam)	[42]
Genus *Terratosyringophilus* Bochkov and Perez, 2002				
*T. geotrygonus* Skoracki & Glowska, 2008	*Geotrygon linearis* * (Prévost)	Columbinae	Neot. (Venezuela)	[37]
*T. longisoma* (Casto, 1979)	*Zenaida asiatica* * (Linnaeus)	Columbinae	Near. (USA)	[31]
“	*Zenaida macroura* (Linnaeus)	Columbinae	Near. (USA)	[37]
Subfamily Picobiinae Johnson & Kethley, 1973				
Genus *Gunabopicobia* Skoracki & Hromada, 2013				
*G. claravis* Kaszewska, Skoracki & Hromada, 2018	*Claravis pretiosa* * (Ferrari-Perez)	Claravinae	Neot. (Colombia)	[26]
*G. geotrygoni* Kaszewska, Skoracki & Hromada, 2018	*Geotrygon linearis* * (Prevost)	Columbinae	Neot. (Venezuela)	[26]
“	*Geotrygon chrysia* Bonaparte	Columbinae	Ocea. (Martinique)	[26]
“	*Geotrygon frenata* (Tschudi)	Columbinae	Neot. (Colombia)	[26]
“	*Geotrygon montana* (Linnaeus)	Columbinae	Neot. (Paraguay)	[26]
*G. masalaje* Kaszewska, Kavetska & Skoracki, 2014	*Ducula bicolor* (Scopoli)	Raphinae	Orie. (Indonesia)	[43]
“	*Ducula rufigaster* (Quoy and Gaimard)	Raphinae	Ocea. (Papua New Guinea)	[43]
“	*Ducula rosacea* (Temminck)	Raphinae	Orie. (Indonesia: Semau Isl.)	[43]
“	*Ducula pistrinaria* Bonaparte	Raphinae	Ocea. (Papua New Guinea)	[43]
“	*Ducula spilorrhoa (Gray)*	Raphinae	Orie. (Indonesia: Semau Isl.)	[43]
“	*Ducula luctuosa* (Temminck)	Raphinae	Ocea. (Papua New Guinea)	[43]
“	*Ptilinopus iozonus* * Gray	Raphinae	Ocea. (Papua New Guinea)	[43]
*G. metriopelia* Kaszewska, Skoracki and Hromada, 2018	*Metriopelia melanoptera* * (Molina)	Claravinae	Neot. (Argentina)	[26]
*G. lathami* Kaszewska, Skoracki and Hromada, 2018	*Leucosarcia melanoleuca* * Gould	Raphinae	Orie. (Indonesia); Ocea. (Papua New Guinea)	[26]
“	*Caloenas nicobarica* (Linnaeus)	Raphinae	Orie. (Indonesia); Ocea. (Papua New Guinea)	[26]
*G. leptotila* Kaszewska, Skoracki & Hromada, 2018	*Leptotila verreauxi* * (Bonaparte)	Columbinae	Neot. (Argentina)	[26]
*G. zumpti* (Lawrence, 1959)	*Columba livia* Gmelin	Columbinae	Near. (USA); Pala. (Poland)	[36,47]
“	*Columba delegorquei* Delegorgue	Columbinae	Afro. (Tanzania)	[26]
“	*Patagioenas picazuro* (Temminck)	Columbinae	Neot. (West Brazil)	[26]
“	*Patagioenas speciosa* Gmelin	Columbinae	Neot. (North Brazil)	[26]
“	*Streptopelia capicola* * (Sundevall)	Columbinae	Afro. (South Africa)	[29]
“	*Streptopelia semitorquata* (Ruppell)	Columbinae	Afro. (Ethiopia)	[26]
“	*Streptopelia senegalensis* (Linnaeus)	Columbinae	Afro.(South Africa)	[41]
“	*Zenaida macroura* (Linnaeus)	Columbinae	Near. (USA)	[28]

Zoogeographical regions: Afro.—Afrotropical, Aust.—Australian, Near.—Nearctic, Neot.—Neotropical, Ocea.—Oceanian, Orie.—Oriental, Pala.—Palaearctic, Pana.—Panamanian, Sa-Arab.—Saharo-Arabian, Si-Jap.—Sino-Jappanese (according to Holt et al. [71]). *—type host; **—host from order Psittaciformes; “— previous species name. Locality established based on the host distribution.

**Table 2:** Host species infested by quill mites with habitat and the index of prevalence (IP) and 95% confidence interval (Sterne’s method).

Host Species		Exa.	Inf.	IP; CI	Mite Species	Habitat
*Caloenas nicobarica* *	Nicobar pigeon	4	2	50 (9.8–90.2)	*G. lathami*	contour
*Chalcophaps indica*	Grey-capped Emerald Dove	23	2	8.7 (1.6–27.8)	*M. chalcophas*	coverts
*Claravis pretiosa*	Blue Ground-dove	10	3	30 (8.7–61.9)	*P. claravis*	coverts
“ *			2	20 (3.7–55.3)	*G. claravis*	contour
*Columba arquatrix*	African Olive-pigeon	4	2	50 (9.8–90.2)	*P. columbae*	under-wings cov.
*Columba delegorguei*	Delegorgue’s Pigeon	7	2	42 (14.9–77.5)	*M. lengai*	under-tail cov.
“ *			1	14.3 (0.7–55.4)	*G. zumpti*	contour
*Columba guinea*	Speckled Pigeon	4	2	50 (9.8–90.2)	*P. columbae*	under-wings cov.
*Columba leucomela*	White-headed Pigeon	1	1	100 (5.0–100)	*P. leucomela*	-
*Columba leuconota*	Snow Pigeon	1	1	100 (5.0–100)	*P. columbae*	-
*Columba livia*	Rock Pigeon	20	1	5 (0.3–24.4)	*P. columbae*	contour
“		NA	NA	-	*G. zumpti*	contour
“		1	1	100 (5.0–100)	*M.zenadourae*	covert
“		NA	NA	-	*M. columbicus*	secondaries
*Columba oenas*	Stock Dove	NA	NA	-	*M. columbicus*	secondaries
“		9	1	11.1 (0.6–44.4)	*P. columbae*	under-wings cov.
*Columba palumbus*	Common Wood-Pigeon	20	1	5 (0.3–24.4)	*M. columbicus*	tail cov.
“		20	1	5 (0.3–24.4)	*P. columbae*	covert
*Columba trocaz*	Madeira laurel Pigeon	1	1	100 (5.0–100)	*P. columbae*	under wing cov.
*Columbina minuta*	Plain-breasted Ground-Dove	4	2	50 (9.8–90.2)	*P.mucuya*	contour
*Columbina passerina*	Common Ground-Dove	15	2	13.3 (2.4–39.7)	*P.mucuya*	secondaries
*Columbina talpacoti*	Ruddy Ground-Dove	32	8	25 (12.2–42.3)	*P.mucuya*	under-wings cov
*Columbina squammata*	Scaled Dove	11	1	9.1 (0.5–40.5)	*P.mucuya*	tertials
*Ducula bicolor* *	Pied Imperial-Pigeon	2	2	100 (22.4–100)	*G. masalaje*	contour
*Ducula luctuosa* *	Silver-tipped Imperial-Pigeon	1	1	100 (22.4–100)	*G. masalaje*	contour
“		1	1	100 (22.4–100)	*P.lature*	covert
*Ducula pistrinaria* *	Island Imperial-Pigeon	2	1	50 (2.5–97.5)	*G. masalaje*	contour
*Ducula rosacea* *	Pink-headed Imperial-Pigeon	1	1	100 (22.4–100)	*G. masalaje*	contour
*Ducula rufigaster* *	Purple-tailed Imperial-Pigeon	1	1	100 (22.4–100)	*G. masalaje*	contour
*Ducula spilorrhoa* *	Torresian Imperial-Pigeon	1	1	100 (5.0–100)	*G. masalaje*	contour
“		1	1	100 (5.0–100)	*P.lature*	-
*Gallicolumba luzonica*	Luzon Bleeding-heart	2	1	50 (2.5–97.5)	*M. turacoenas*	contour
*Geopelia cuneata*	Diamond Dove	2	1	50 (2.5–97.5)	*P. geopelis*	covert
*Geopelia placida*	Peaceful Dove	2	1	50 (2.5–97.5)	*P. geopelis*	contour
*Geopelia striata*	Zebra Dove	13	5	38.5 (16.6–65.8)	*P. geopelis*	covert
*Geotrygon chrysie* *	Key West Quail-Dove	1	1	100 (22.4–100)	*G. geotrygoni*	contour
*Geotrygon chiriquensis*	Chiriqui Quail-Dove	1	1	100 (5–100)	*P. columbae*	under-tail cov.
*Geotrygon frenata*	White-throated Quail-Dove	3	1	33 (1.7–86.5)	*M. zenadourae*	-
“ *			1	33 (1.7–86.5)	*G. geotrygoni*	contour
*Geotrygon linearis* *	Lined Quail-Dove	8	1	12.5 (0.6–50)	*G. geotrygoni*	contour
			1	12.5 (0.6–50)	*T. geotrygonus*	primaries
*Geotrygon montana* *	Ruddy Quail-Dove	8	2	25 (4.6–63.5)	*G. geotrygoni*	contour
“			1	12.5 (0.6–50)	*P. montanus*	under tail cov.
*Leptotila verreauxi*	White-tipped dove	24	1	4.2 (0.2–20.4)	*M. zenadourae*	under-tail cov.
“ *			1	4.2 (0.2–20.4)	*G. leptotila*	contour
*Leptotila rufaxilla*	Gray-fronted Dove	10	2	20 (3.7–55.3)	*M. zenadourae*	under-tail cov.
*Leucosarcia melanoleuca* *	Wonga Pigeon	1	1	100 (5–100)	*G. lathami*	contour
*Macropygia amboinensis*	Amboyna Cuckoo-Dove	6	1	16.7 (0.9–58.9)	*M. turacoenas*	-
*Macropygia phasianella*	Brown Cuckoo-Dove	14	3	21.4 (6.1–50)	*M. turacoenas*	under and upper-tail cov.
*Macropygia unchall*	Barred Cuckoo-Dove	3	2	66.7 (13.5–98.3)	*M. turacoenas*	under-tail cov.
*Metriopelia ceciliae*	Bare-faced Ground-Dove	2	1	50 (2.5–97.5)	*P. mucuya*	secondaries, covert
*Metriopelia melanoptera* *	Black-winged Ground-Dove	8	1	12.5 (0.6–50)	*G. metriopelia*	contour
*Ocyphaps lophotes*	Crested Pigeon	1	1	100 (5–100)	*P. geopelis*	small covert under-tail cov.
*Oena capensis*	Namaqua Dove	17	5	29.4 (12.4–54.4)	*P. claravis*	under-tail cov.
*Patagioenas fasciata*	Band-tailed pigeon	1	1	100 (5.0–100)	*P. patagioenas*	upper-tail cov.
*Patagioenas picazuro*	Picazuro Pigeon	16	2	12.5 (2.3–37.2)	*M. zenadourae*	under-wing cov.
“ *			1	6.2 (0.3–30.5)	*G. zumpti*	contour
*Patagioenas speciosa*	Scaled Pigeon	8	1	12.5 (0.6–50)	*P. columbae*	-
“ *			1	12.5 (0.6–50)	*G. zumpti*	contour
“			1	12.5 (0.6–50)	*P. patagioenas*	coverts
*Ptilinopus iozonus* *	Orange-Bellied Fruit Dove	4	1	25 (1.3–75.1)	*G. masalaje*	contour
*Ptilinopus jambu*	Jambu Fruit-Dove	5	1	20 (1–65,7)	*P. lature*	coverts
*Ptilinopus magnificus*	Wompoo Fruit-Dove	17	2	11.8 (2.1–35)	*M. ptilinopus*	under-tail cov.
*Ptilinopus melanospilus*	Black-naped Fruit-Dove	3	2	66.7 (13.5–98.3)	*P. lature*	coverts
*Ptilinopus regina*	Rose-crowned Fruit-Dove	4	1	25 (1.3–75.1)	*P. lature*	coverts
*Ptilonopus rivoli*	White-bibbed Fruit-Dove	1	1	100 (5–100)	*M. ptilinopus*	under-tail cov.
*Streptopelia decaocto*	Eurasian Collared-Dove	12	2	16.7 (3–45.7)	*P. columbae*	secondaries
*Streptopelia capicola*	Ring-Necked Dove	NA	NA	-	*G. zumpti*	contour
*Streptopelia orientalis*	Oriental Turtle-Dove	22	2	9.1 (1.6–29.1)	*M. lengai*	under-tail cov.
*Streptopelia semitorquata*	Red-eyed Dove	21	1	4.8 (0.2–23.3)	*M. lengai*	rectrices
“ *			1	4.8 (0.2–23.3)	*G. zumpti*	contour
“			3	14.3 (4–35.4)	*P. columbae*	coverts
*Streptopelia turtur*	European Turtle-Dove	30	4	13.3 (4.7–29.8)	*P. columbae*	contour under-tail cov.
*Treron waalia*	Bruce’s Green-Pigeon	1	1	100 (5.0–100)	*M. columbicus*	covert
*Turacoena manadensis*	White-faced Cuckoo-Dove	4	1	25 (1.3–75.1)	*M. turacoenas*	under tail cov
*Turacoena modesta*	Black Cuckoo-Dove	5	1	20 (1–65.7)	*M. turacoenas*	under tail cov
*Turtur chalcospilos*	Emerald-spotted Wood-Dove	13	1	7 (0.4–34.2)	*M. tympanistria*	coverts
*Turtur tympanistria*	Tambourine Dove	12	2	16.7 (3–45.7)	*M. tympanistria*	rectrices
*Zenaida asiatica*	White-winged Dove	NA	NA	-	*M. zenadourae*	-
“		NA	NA	-	*T. longisoma*	-
*Zenaida auriculata*	Eared Dove	NA	NA	-	*M. zenadourae*	-
*Zenaida macroura*	Mourning Dove	1	1	100 (5–100)	*M. zenadourae*	coverts
“		NA	NA	-	*T. longisoma*	primaries

Exa.—number of individual host species examined during study; Inf.—number of individual host species, infected by quill mites; IP—prevalence index given in (%); CI—confidence interval (Sterne method); NA—infected hosts species, but prevalence index was unknown. *—type host; “— previous species name.

**Table 3:** Host specificity of quill mite species of the subfamily Syringophilinae with the value of d’ index.

Specificity	d’	Quill Mites	Hosts Spectrum
Monoxenous	0.2	*Meitingsunes adwelles*	*Geotrygon frenata*
	1	*Meitingsunes chalcophas*	*Chalcophaps indica*
	1	*Peristerophila leucomela*	*Columba leucomela*
	0.75	*Psittaciphilus montanus*	*Geotrygon montana*
	0.5	*Terratosyringophilus geotrygonus*	*Geotrygon linearis*
Oligoxenous	1	*Meitingsunes tympanistria*	*Turtur chalcospilos*
			*Turtur tympanistria*
	0.77	*Psittaciphilus patagioenas*	*Patagioenas fasciata*
			*Patagioenas speciosa*
	0.46	*Terratosyringophilus longisoma*	*Zenaida asiatica*
			*Zenaida macroura*
	1	*Meitingsunes ptilinopus*	*Ptilinopus magnificus*
			*Ptilinopus rivoli*
Mesostenoxenous	0.9	*Meitingsunes lengai*	*Columba delegorguei*
			*Streptopelia orientalis*
			*Streptopelia semitorquata*
	0.92	*Meitingsunes zenadourae*	*Columba livia*
			*Geotrygon frenata*
			*Leptotila verreauxi*
			*Leptotila rufaxilla*
			*Patagioenas picazuro*
			*Zenaida asiatica*
			*Zenaida auriculata*
			*Zenaida macroura*
	0.95	*Peristerophila columbae*	*Columba arquatrix*
			*Columba guinea*
			*Columba livia*
			*Columba oenas*
			*Columba palumbus*
			*Columba leuconota*
			*Columba trocaz*
			*Geotrygon chiriquensis*
			*Patagioenas speciosa*
			*Streptopelia capicola*
			*Streptopelia decaocto*
			*Streptopelia orientalis*
			*Streptopelia semitorquata*
			*Streptopelia tranquebarica*
			*Streptopelia turtur*
	1	*Peristerophila geopelis*	*Geopelia cuneata*
			*Geopelia placida*
			*Geopelia striata*
			*Ocyphaps lophotes*
	0.86	*Peristerophila lature*	*Ducula luctuosa*
			*Ducula spilorrhoa*
			*Ptilinopus melanospilus*
			*Ptilinopus porphyreus*
			*Ptilinopus regina*
Metastenoxenous	1	*Meitingsunes turacoenas*	*Gallicolumba luzonica*
			*Macropygia amboinensis*
			*Macropygia phasianella*
			*Macropygia unchall*
			*Turacoena manadensis*
			*Turacoena modesta*
	0.92	*Peristerophila claravis*	*Claravis pretiosa*
			*Oena capensis*
	0.78	*Meitingsunes columbicus*	*Columba livia*
			*Columba oenas*
			*Columba palumbus*
			*Treron waalia*
Polixenous	0.98	*Peristerophila mucuya*	*Columbina minuta*
			*Columbina passerina*
			*Columbina squammata*
			*Columbina talpacoti*
			*Metriopelia ceciliae*
			*Metriopelia melanoptera*
			*Streptopelia decaocto*
			*Brotogeris versicolurus* *
			*Psilopsiagon aymara* *
			*Trichoglossus haematodus* *

d’—index measured specialization at species level; *—hosts species belonging to order Psittaciformes.

**Table 4:** Host specificity of quill mite species the subfamily Picobiinae with the value of d’ index.

Specificity	d’	Quill Mites	Hosts Spectrum
Monoxenous	0.78	*Gunabopicobia claravis*	*Claravis pretiosa*
	0.85	*Gunabopicobia leptotila*	*Leptotila verreauxi*
	0.98	*Gunabopicobia metriopelia*	*Metriopelia melanoptera*
Oligoxenous	0.92	*Gunabopicobia geotrygoni*	*Geotrygon chrysia*
			*Geotrygon frenata*
			*Geotrygon linearis*
			*Geotrygon montana*
Mesostenoxenous	1	*Gunabopicobia lathami*	*Caloenas nicobarica*
			*Leucosarcia melanoleuca*
	0.9	*Gunabopicobia masalaje*	*Ducula bicolor*
			*Ducula luctuosa*
			*Ducula pistrinaria*
			*Ducula rosacea*
			*Ducula rufigaster*
			*Ducula spilorrhoa*
			*Ptilinopus iozonus*
	0.66	*Gunabopicobia zumpti*	*Columba delegorguei*
			*Columba livia*
			*Patagioenas picazuro*
			*Patagioenas speciosa*
			*Streptopelia capicola*
			*Streptopelia semitorquata*
			*Streptopelia senegalensis*
			*Zenaida macroura*

d’—index measured specialization on species level.

**Table 5:** Host species infested by two or more syringophilid species with notation of the habitat preference; P—Picobiinae; S—Syringophilinae.

Hosts	Quill Mites	Subfamily	Niche
*Claravis pretiosa*	*Gunabopicobia claravis*	P	contour
	*Peristerophila claravis*	S	covert
*Columba palumbus*	*Meitingsunes columbicus*	S	secondaries
	*Peristerophila columbae*	S	covert
*Ducula spilorrhoa*	*Gunabopicobia masalaje*	P	contour
	*Peristerophila lature*	S	covert
*Ducula luctuosa*	*Gunabopiconia masalaje*	P	contour
	*Peristerophila lature*	S	covert
*Geotrygon frenata*	*Gunabopicobia geotrygoni*	P	contour
	*Meitingsnes zenadourae*	S	under-wing covert
*Geotrygon montana*	*Gunabopicobia geotrygoni*	P	contour
	*Psittaciphilus montanus*	S	under-tail covert
*Leptotila verreauxi*	*Gunabopicobia leptotila*	P	contour
	*Meitingsunes zenadoure*	S	under tail-covert
*Metriopelia melanoptera*	*Gunapopicobia metriopelia*	P	contour
	*Peristerophila mucuya*	S	covert
*Patagioenas picazuro*	*Gunabopicobia zumpti*	P	contour
	*Meitingsunes zenadourae*	S	under-wing covert
*Patagioenas speciosa*	*Gunabopicobia zumpti*	P	contour
	*Peristerophila columbae*	S	covert
	*Psittaciphilus patagioenas*	S	covert
*Streptopelia semitorquata*	*Gunabopicobia zumpti*	P	contour
	*Meitingsunes lengai*	S	rectrices
	*Peristerophila columbae*	S	covert

Subfamily of the family Syringophilidae: (P)—Picobiinae, (S)—Syringophilidae.

**Table 6:** Distribution of syringophilid associated with birds from order Columbiformes in zoogeographical regions.

	Zoogeographic Regions								
Quill Mites Species	Neot.	Near.	Pana.	Pala.	Sa-Ara.	Afro.	Orie.	Ocean.	Austr.
*M. aldwelles*									
*M. columbicus*									
*M. chalcophas*									
*M. ptilinopus*									
*M. lengai*									
*M. turacoenas*									
*M. tympanistria*									
*M. zenadourae*									
*P. columbae*									
*P. claravis*									
*P. geopelis*									
*P. lature*									
*P. leucomela*									
*P. mucuya*									
*P. montanus*									
*P. patagioenas*									
*T. geotrygonus*									
*T. longisoma*									
*G. claravis*									
*G. geotrygoni*									
*G. masalaje*									
*G. metriopelia*									
*G. lathami*									
*G. leptotila*									
*G. zumpti*									

Zoogeographical regions: Afro.—Afrotropical, Aust.—Australian, Near.—Nearctic, Neot.—Neotropical, Ocea.—Oceanian, Orie.—Oriental, Pala.—Palaearctic, Pana.—Panamanian, Sa-Arab.—Saharo-Arabian, Si-Jap.—Sino-Japanese (according to Holt et al. [71]).

## Data Availability

Data is available upon request from the corresponding authors.

## Supplementary Materials

The following are available online at https://www.mdpi.com/article/10.3390/ani11123392/s1, Table S1. The degree of Syringophilidae examination for each Columbiformes subfamily; Figure S1. A network matrix; Figure S2. Characters; Figure S3. Data matrix.
