# Supplementary material for: Global Studies of the Host-Parasite Relationships between Ectoparasitic Mites of the Family Syringophilidae and Birds of the Order Columbiformes
Source: Animals (Basel). 2021 Nov 27;11(12):3392. doi: 10.3390/ani11123392 (PMC8697884; doi:10.3390/ani11123392)
Supplement: Supplementary file 1 [file animals-11-03392-s001.zip › animals-1465214-supplementary.pdf]

Table S1. The degree of Syringophilidae examination for each Columbiformes subfamily

Host subfamily	Host genus	Species	Examined	Quill mites genus assocoaited with Columbiformes species				
				Meitingsunes	*Psittaciphilus*	*Peristerophila*	*Terratosyringophilus*	*Gunabopicobia*
**Columbinae**	*Columba*	36	(10) 27%			**8**		**2**
	Ectopistes	1	-					
	Geotrygon	17	(6) 35%	**1**	**1**	**1**	**1**	**3**
	Leptotila	10	(3) 30%	**2**				**1**
	Leptotrygon	1	(1) 100%					
	Macropygia	15	(5) 33%	**3**				
	Patagioenas	17	(7) 41%	**1**	**2**	**1**		
	Reinwardtoena	3	-					
	Streptopelia	17	(9) 52%	**2**		**3**		**2**
	Turacoena	3	(2) 66%	**2**				
	Zenaida	7	(6) 85%	**5**			**2**	
**Claraviinae**	*Claravis*	1	(1) 100%			**1**		**1**
	Columbina	9	(5) 55%			**4**		
	Metriopelia	4	(2) 50%			**1**		**1**
	Paraclaravis	2	(1) 50%					
	Uropelia	1	-					
**Raphinae**	*Alectroenas*	5	-					
	Alopecoenas	13	-					
	Caloenas	2	(1) 50%					**1**
	Chalcophas	3	(2) 66%	**1**				
	Cryptophaps	1	-					
	Didunculus	1	-					
	Drepanoptila	1	-					
	Ducula	39	(11) 28%			**2**		**6**
	Gallicolumba	7	(2) 28%	**1**				
	Geopelia	5	(3) 60%			**3**		
	Geophaps	3	-					

Goura	4	-					
Gymnophaps	4	(1) 25%					
Hemiphaga	2	-					
Henicophaps	2	(2) 100%					
Leptotrygon	1	(1) 100%					
Leucosarcia	1	(1) 100%					**1**
Lopholaimus	1	-					
Microgoura	1	-					
Nesoenas	4	-					
Ocyphaps	1	(1) 100%			**1**		
Oena	1	(1) 100%			**1**		
Otidiphaps	1	(1) 100%					
Petrophassa	2	-					
Pezophaps	1	-					
Phapitreron	4	-					
Phaps	3	-					
Ptilinopus	55	(14) 25%	**2**		**3**		**1**
Raphus	1	-					
Starnoenas	1	-					
Treron	29	(12) 41%	**1**				
Trugon	1	-					
Turtur	5	(2) 40%	**2**				

Figure S1. A network matrix

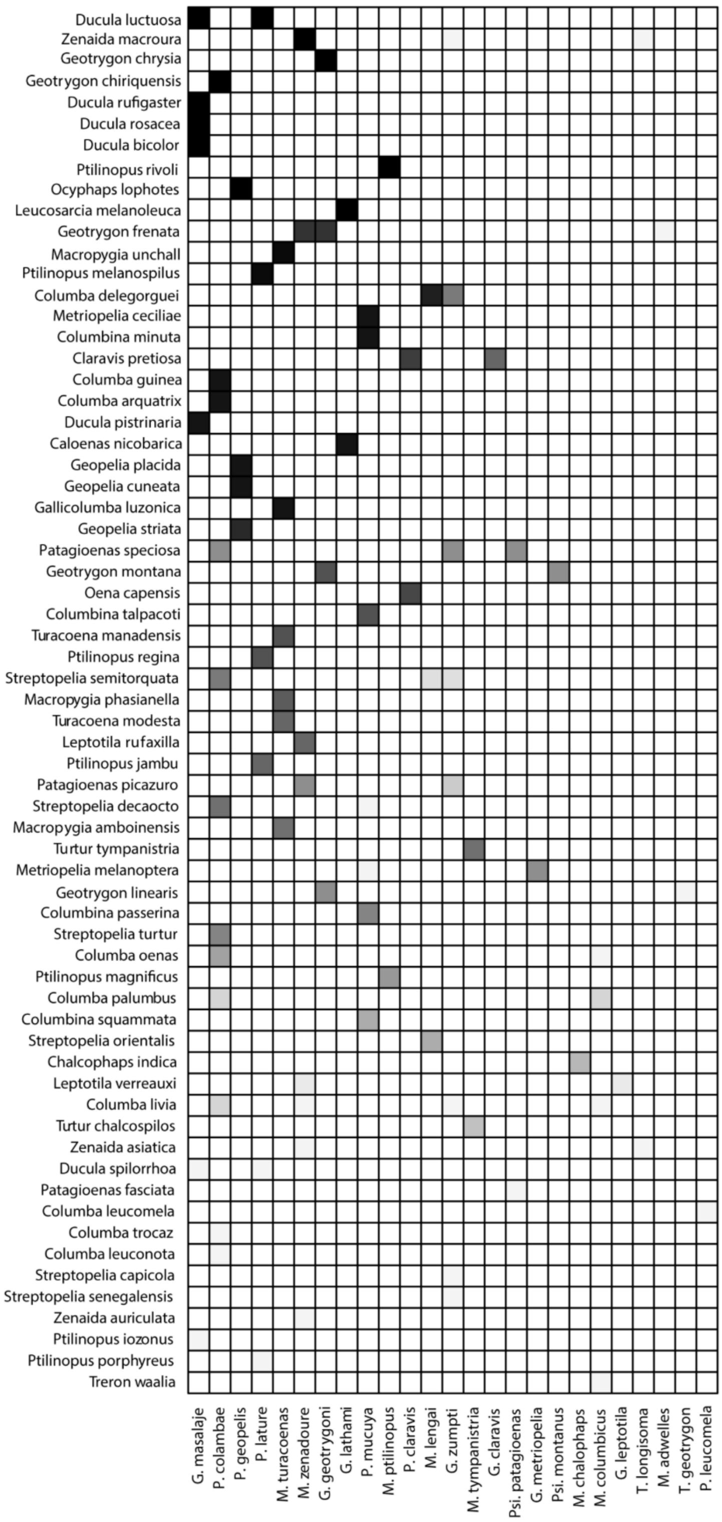

Figure S2. Characters

1. Posterior part of stylophore: 0. rounded; 1. strongly constricted.
2. Large sausage-like hypostomal structures: 0. absent; 1. present.
3. Peritremes - shape: 0. M-shaped; 1. U-shaped.
4. Distal tip of chelicerae: 0. edentate; 1. small teeth; 2. large teeth.
5. vi setae: 0. present; 1. absent.
6. Position of setae ve and si: 0. ve situated anterior to si; 1. ve and si situated at same transverse level.
7. Setae g1: 0. present; 1. absent.
8. Ornamentation of dorsal setae: 0. absent; 1. present.
9. Pocket-like structures: 0. absent; 1. present.
10. Leg thickness : 0. I thicker than II-IV; 1. I and II thicker than III and IV; 2. subequal.
11. Apodemes I: 0. divergent; 1. parallel.
12. Apodemes I and II fusion: 0. fused; 1. not fused; 2. fusion indistinct (adjoining).
13. Apodemes III-IV: 0. present; 1. absent.
14. Setae vs on tarsus II: 0. present; 1. absent.
15. Prorals setae p' and p": 0. rod-like, with rounded or serrate apex; 1. fan-like; 2. multiserrate.
16. Setae dF on femora II: 0. present; 1. absent.
17. Setae vF on femora III: 0. present; 1. absent.
18. Setae dF on femora III: 0. present; 1. absent.
19. Setae l'R on trochanters I: 0. present; 1. absent.
20. Bimorphism: 0. absent; 1. present.
21. Basal part of gnathosomanot: 0. not submerged; 1. deeply submerged.
22. Palpal tibia and tarsus: 0. separated; 1. fused.
23. Claw-like seta of palp: 0. present; 1. absent.
24. Palpal apex: 0. rounded; 1. truncated.
25. Setae e1 presence: 0. present; 1. absent.
26. Setae ps3 presentce: 0. present; 1. absent.

Figure S3. Data matrix.

Meitingsunes_zenadourae	1001100000111211100111011
Psittaciphilus_montanus	1001110010111211100111011
Peristerophila_nestoriae	0101100001011211101111011
Terratosyringophilus_geotrygonus	0101100001111210101111011
Gunabopicobia_claravis	0010001101100001010111111
Cheyletus	00000000000000000000000000
Metacheyletoides	00000000000000000000000000
